# Platelet Counts and Liver Enzymes after Bariatric Surgery

**DOI:** 10.1155/2013/567984

**Published:** 2013-02-20

**Authors:** Hans-Erik Johansson, Arvo Haenni, Björn Zethelius

**Affiliations:** ^1^Section of Geriatrics, Department of Public Health and Caring Sciences, Uppsala University, Uppsala Science Park, 75185 Uppsala, Sweden; ^2^Medical Products Agency, Uppsala, Sweden

## Abstract

*Background*. Obesity is characterized by liver steatosis, chronic inflammation, and increased liver enzymes, that is, gamma-glutamyltransferase (GGT) and alanine aminotransferase (ALT), markers for nonalcoholic fatty liver disease (NAFLD) and liver fat content. Increased platelet counts (PCs) are associated with inflammatory conditions and are a valuable biomarker of the degree of fibrosis in NAFLD. We investigated alterations in PC, GGT, and ALT after biliopancreatic diversion with duodenal switch (BPD-DS) and Roux-en-Y gastric bypass (RYGBP). *Methods*. Ten morbidly obese patients (body mass index, BMI: 53.5 ± 3.8 kg/m^2^) who underwent BPD-DS were evaluated preoperatively (baseline) and 1 year (1st followup) and 3 years (2nd followup) after surgery and compared with 21 morbidly obese patients (BMI: 42.3 ± 5.2 kg/m^2^) who underwent RYGBP. *Results*. Over the 3 years of followup, changes in BPD-DS and RYGBP patients (BPD-DS/RYGBP) were as follows: BMI (−44%/−24%), GGT (−63%/−52%), and ALT (−48%/−62%). PC decreased (−21%) statistically significantly only in BPD-DS patients. *Conclusions*. Morbidly obese patients treated by RYGBP or BPD-DS show sustained reductions in BMI, ALT, and GGT. The decrease in PC and liver enzymes after BPD-DS may reflect a more pronounced decrease of liver-fat-content-related inflammation and, as a result, a lowered secondary thrombocytosis.

## 1. Introduction

Bariatric surgery has become an effective treatment for obesity, also reducing the onset of type 2 diabetes mellitus (T2DM) [1] as well as inducing remission [2] and reducing cardiovascular mortality and mortality in general [3, 4]. 

Obesity is a chronic condition characterized by elevated inflammatory markers [5–7] and is associated with nonalcoholic fatty liver disease (NAFLD) [8, 9]. Serum gamma-glutamyltransferase (GGT) and alanine aminotransferase (ALT) are markers of NAFLD and of liver fat content [10, 11]. Increased platelet counts have been observed in conditions with chronic inflammation as well as in obesity, probably due to secondary thrombocytosis [12–14]. In more advanced stages of NAFLD, with portal hypertension and splenomegaly, reduced platelet counts have been observed [15]. There is a linear association between decreased platelet counts and increased fibrosis in the histopathology of liver biopsies, which may indicate that platelet counts might be an important biomarker of the degree of fibrosis in NAFLD patients. Platelet count is a simple, easy to perform, cost-effective, and accurate surrogate marker for predicting fibrosis severity in NAFLD patients [16].

Bariatric surgery improves steatosis and fibrosis in patients with morbid obesity NAFLD [17], and adjustable gastric banding in a previous study has been related to a nonsignificant trend of lowered platelet counts [18]. Regarding RYGBP, one study reports a significant reduction in platelet counts 1 year after surgery [14]. Data after longer periods of followup, after RYGBP as well as BPD-DS surgeries, are lacking.

The aim of this study was to assess changes, if any, in morbidly obese patients treated with BPD-DS and RYGBP, from baseline, that is, before surgery, to followups 1 year and 3 years after surgery, with regard to platelet counts and serum concentrations of GGT and ALT.

## 2. Material and Methods

### 2.1. Patients

Ten morbidly obese patients who had undergone BPD-DS surgery (five men and five women), all Caucasians, free from established diabetes, were recruited from the Outpatient Clinic of Obesity Care, Uppsala University Hospital, Uppsala, Sweden [19]. Data from the BPD-DS group were compared to that of a morbidly obese group (*n* = 21; three men and eighteen women), all free from established diabetes and had undergone RYGBP [20]. Patients were investigated preoperatively (baseline) and then 1 year (1st followup) and 3 years (2nd followup) after BPD-DS and RYGBP, respectively. The study was approved by the regional ethics review board at Uppsala University.

### 2.2. BPD-DS and RYGBP Surgeries Procedures

Roux-en-Y gastric bypass is a procedure that combines restriction and malabsorption. It is considered by many to be the gold standard because of its high level of effectiveness and its durability. More extreme malabsorption accompanies biliopancreatic diversion procedures, commonly performed with a duodenal switch in which a short, distal and common channel length of small intestine severely limits caloric absorption, which induces a greater weight loss than RYGBP does [21]. A very high BMI indicated that BPD-DS procedure was performed instead of RYGBP at our clinic [19, 20].

### 2.3. Test Procedures

All participants underwent physical examination and blood tests for platelet count, GGT, and ALT preoperatively (baseline) and at the 1st and 2nd followups. Blood samples were collected from each patient (following an overnight fast) and were analyzed using routine tests at the Department of Clinical Chemistry at Uppsala University Hospital [19, 20].

### 2.4. Clinical Measurements

Weight (kg) and height (m) were measured on standardized calibrated scales, and BMI (kg/m^2^) was calculated. 

### 2.5. Statistics

All analyses were defined a priori. Results are presented as arithmetic means, with standard deviations. ANOVA was used to investigate trends over the 3 years of followup. Changes between different time points were analyzed using paired *t*-tests. Tests were two tailed, and a *P*  value <0.05 was considered significant. Statistical software JMP 3.2 for PC (SAS Institute Inc., Cary, NC, USA) was used.

## 3. Results

### 3.1. Baseline Data

Patient clinical characteristics at baseline, that is, before RYGBP and BPD-DS surgeries, are shown in Table 1. There were no statistically significant intergroup differences in platelet counts or plasma concentrations of GGT and ALT. Mean weight and BMI were, as expected, higher in the BPD-DS group, selecting the patients for this procedure, and age was lower.

### 3.2. Followup Data at 1 Year (1st Followup) and 3 Years (2nd Followup)

**Figure 1:**
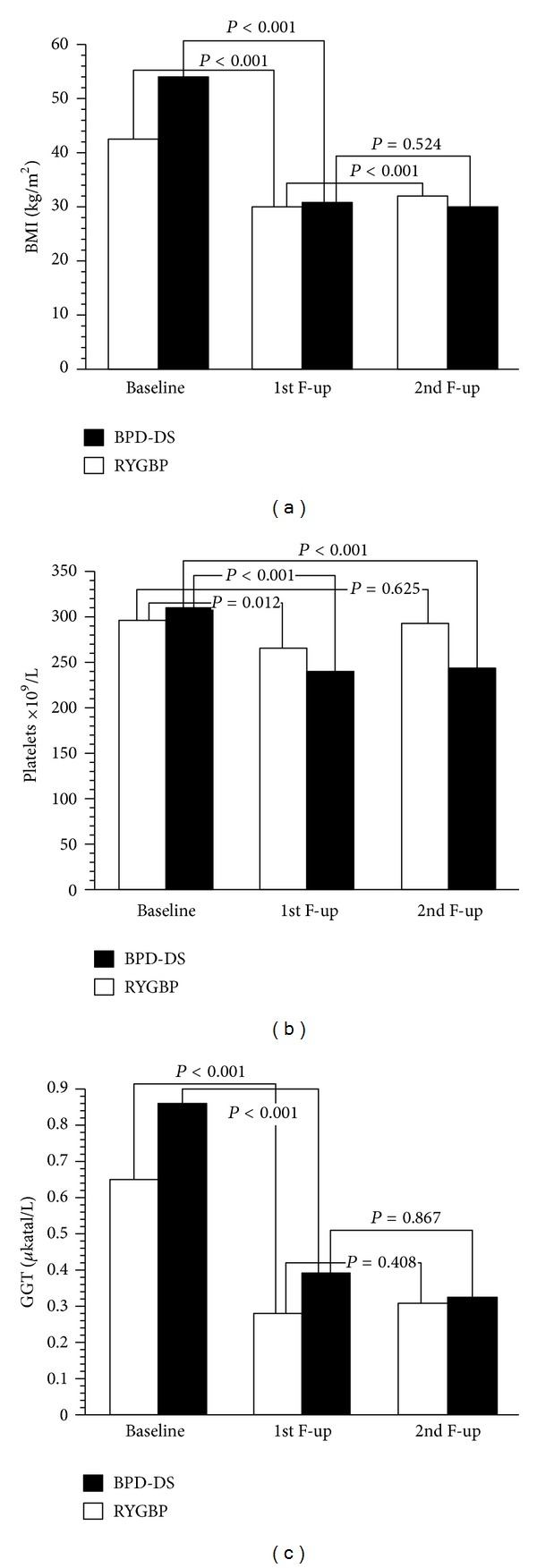
The changes in BMI (a), platelet counts (b), and concentrations of gamma-glutamyltransferase (GGT) (c) are shown at baseline, that is, before surgery, at the 1st followup (1 year), and at the 2nd followup (3 years). Group symbols are as follows: morbidly obese patients treated with Roux-en-y gastric bypass surgery, *white bars*; morbidly obese patients treated with biliopancreatic diversion with duodenal switch, *black bars*. Mean values are shown. Statistical significance is indicated by *P*  values. F-up denotes followup.

BMI decreased by 43% in the BPD-DS group from 53.5 kg/m^2^ at baseline to 30.7 kg/m^2^ at 1st followup (*P* < 0.001) and was unchanged between the 1st and 2nd followups (30.2 kg/m^2^,  *P* < 0.001), as shown in Figure 1(a). In the RYGBP group, BMI was reduced by 30% from 42.3 kg/m^2^ at baseline to 29.7 kg/m^2^ at the 1st followup (*P* < 0.001) but was 32.1 kg/m^2^ at the 2nd followup (*P* < 0.001), implying a 6% gain (gain over the baseline BMI) between the 1st and 2nd followups (*P* < 0.001).

Platelet counts were reduced by 22% in the BPD-DS group from 308 × 10^9^/L at baseline to 240 × 10^9^/L at the 1st followup (*P* < 0.001) and were unchanged at the 2nd followup (244 × 10^9^/L,  *P* < 0.001), as shown in Figure 1(b). In the RYGBP group, platelet counts were reduced by 10% from 297 × 10^9^/L at baseline to 266 × 10^9^/L at the 1st followup (*P* = 0.012) but were 292 × 10^9^/L at the 2nd followup (*P* = 0.687), implying a 9% increase between the 1st and 2nd followups (*P* = 0.024).

GGT was markedly lowered by 55% in the BPD-DS group from 0.87 *μ*katal/L at baseline to 0.39 *μ*katal/L at the 1st followup (*P* = 0.01) and was 0.32 *μ*katal/L at the 2nd followup (*P* < 0.001), but this further decrease was not significant (*P* = 0.867), as shown in Figure 1(c). In the RYGBP group, GGT was lowered by 57% from 0.65 *μ*katal/L at baseline to 0.28 *μ*katal/L at the 1st followup (*P* < 0.001), with no further alteration at the 2nd followup (0.31 *μ*katal/L, *P* < 0.001).

ALT was reduced by 46% in the BPD-DS group from 0.79 *μ*katal/L at baseline to 0.43 *μ*katal/L at the 1st followup (*P* = 0.004) and was unchanged at the 2nd followup (0.41 *μ*katal/L, *P* = 0.004). In the RYGBP group, ALT was lowered by 45% from 0.62 *μ*katal/L at baseline to 0.34 *μ*katal/L at the 1st followup (*P* < 0.001) and was 0.24 *μ*katal/L at the 2nd followup (*P* < 0.001), implying a further decrease by 16% between the 1st and 2nd followups (*P* = 0.02).

## 4. Discussion

The main findings in this study were that liver enzymes, GGT and ALT, markedly decreased over time after both RYGBP and BPD-DS surgeries, but platelet counts only decreased significantly after BPD-DS. The alteration in platelet counts showed a somewhat different pattern after RYGBP, with a reduction at the 1st followup but no significant change at the 2nd followup. It might be speculated that the sustained reduction in platelet counts may indicate a long-term improvement in the inflammation of the liver and a more pronounced decrease of liver-fat-content-related inflammation in obese patients treated by BPD-DS compared to RYGBP. Platelets vary daily and are depending on a variety of issues such as ethnicity, age, and gender. However, longitudinal studies demonstrate considerable stability of steady-state platelet counts. Buckley et al. have showed in their analysis of serial platelet counts from 3,789 subjects that the repeatability of the platelet count is very high [22]. Obesity is an inflammatory condition [23, 24] and a major risk factor for the development of NAFLD and liver disease. In obese patients, ultrasonographic examinations as well as liver biopsies have revealed that NAFLD is very common [17]. As it is difficult to perform biopsies in all NAFLD patients, biomarkers are warranted to predict prognosis and to optimize treatments. It has been reported that a lowered GGT may best predict improvements in inflammation and fibrosis in the hepatocytes in NAFLD, which are two major prognostic features in this condition, whereas changes in aminotransferase concentrations did not predict change in steatosis [11]. Furthermore, increased numbers of platelets are observed in conditions with low-grade inflammation, such as obesity, although the platelet counts are within normal ranges [25]. Overweight, obese, and morbidly obese females have significantly elevated platelet counts compared with normal-weight females and male subgroups [25]. The gender difference in platelets might be due to the higher body fat mass in females. Higher platelet counts are associated with more adverse clinical outcomes in patients with myocardial infarction and stroke [25]. Obesity is also associated with platelet dysfunction, increased adhesiveness, and activation [26–28]. In more severe states of NAFLD with fibrosis, a consumption of thrombocytes are observed [29]. In a recent study, Yoneda et al. used liver biopsies to evaluate the clinical usefulness of measuring platelet counts for predicting the severity of liver fibrosis in 1,048 patients with NAFLD [16]. They suggest platelet count to be a major “ideal” biomarker. Bariatric surgery reverses or improves NAFLD in many cases. There is a lack of data on platelet count changes after BPD-DS. Our data show a sustained reduction in platelet counts over time after BPD-DS, probably induced by a more pronounced weight loss than after RYGBP and possibly a more pronounced decrease of liver inflammation. One year after RYGBP, a significant reduction in platelet counts was observed, which is in accordance with 1 year data from Dallal et al. [14], but the reduction was not sustained at the 2nd followup, 3 years after surgery.

There are several limitations in the present study such as the small number of patients and the lack of a morbidly obese control group followedup over 3 years. However, such patients can be logistically difficult to follow for long term followups. The BPD-DS group was significantly younger than the RYGBP group, but no differences were observed between the two groups at baseline in platelet counts, GGT or ALT. Body fat content and liver fat content, measured by imaging techniques such as dual energy X-ray absorptiometry or ultrasonography, would have been warranted to investigate if and how different fat distribution might influence the variables analyzed in this study. 

In conclusion, morbidly obese patients treated with RYGBP and BPD-DS show a marked and sustained decrease in GGT and ALT. A significant reduction in platelets, a marker for inflammation and fibrosis in NAFLD, was observed in both groups after 1 year but only in BPD-DS over time, which may indicate improvements in general inflammatory status and particularly steatohepatitis. However, extended studies are needed to confirm our findings.

## Figures and Tables

**Table 1:** Clinical characteristics at baseline for patients, preoperatively to RYGBP and BPD-DS surgery and 1 and 3 years of followup after surgery.

	RYGBP baseline	BPD-DS baseline	RYGBP	BPD-DS	RYGBP	BPD-DS	*P* for difference at baseline
			1 year	1 year	3 years	3 years	
Gender (women/men)	18/3	5/5	18/3	5/5	18/3	5/5	—
Age (years)	45.7 (9.7)	37.0 (6.5)	—	—	—	—	0.017
Height (cm)	168.4 (6.2)	173.0 (10.1)	—	—	—	—	0.119
Weight (kg)	120.0 (16.4)	161.3 (26.7)	84.0 (13.6)	92.9 (21.4)	90.8 (16.0)	91.9 (25.8)	<0.001
BMI (kg/m^2^)	42.3 (5.2)	53.5 (3.8)	29.7 (4.6)	30.7 (4.6)	32.1 (5.3)	30.2 (5.0)	<0.001
Platelet counts (×10^9^/L)	297 (58)	308 (55)	266 (44)	240 (40)	292 (64)	244 (56)	0.645
P-GGT (*μ*katal/L)	0.65 (0.42)	0.87 (0.71)	0.28 (0.19)	0.39 (0.30)	0.31 (0.24)	0.32 (0.30)	0.334
P-ALT (*μ*katal/L)	0.62 (0.25)	0.79 (0.39)	0.34 (0.18)	0.43 (0.18)	0.24 (0.10)	0.41 (0.13)	0.154

Data shown are arithmetic means (±SD).

BMI: body mass index, GGT: gamma-glutamyltransferase, ALT: alanine aminotransferase, and P: plasma.
